# Survey of parasitic fauna of different ornamental freshwater fish species in Iran

**Published:** 2015-03-15

**Authors:** Milad Adel, Fatemeh Ghasempour, Hamid Reza Azizi, Mohamad Hadi Shateri, Ahmad Reza Safian

**Affiliations:** 1 *Department of Aquatic Animal Health and Diseases, Caspian Sea Ecology Research Center, Sari, Iran; *; 2 *DVMGraduate, Faculty of Veterinary Medicine, Shahrekord University, Shahrekord, Iran; *; 3 *Department of Pathobiology, Faculty of Veterinary Medicine, Shahrekord University, Shahrekord, Iran.*

**Keywords:** Iran, Ornamental Fish, Parasites

## Abstract

Parasitic diseases are harmful and limiting factors in breeding and rearing ornamental fish industry. In this study, 400 apparently healthy ornamental fishes from five species (each species 80 specimens) including: Goldfish (*Carassius auratus*), guppy (*Poecilia reticulate*), angelfish (*Pterophyllum scalare*)*, *discus (*Symphsodon discus*) and sailfin mollies (*Poecilia latipinna*) was obtained from a local ornamental fish farm in the north of Iran during 2011 to 2012. The primary purpose of this study was to determine the parasitic infections of aquarium fish in Iran. For this purpose, fish were first examined for ectoparasites using wet mount under a light microscope. Then, the alimentary ducts of fish were observed under light and stereo microscope. In survey of different infection rates for different parasitic infections in examining fish: *Dactylogyrus *sp.,* Gyrodactylus *sp., *Ichthyophthirius multifiliis* *Trichodina reticulata*, *Capillaria *sp. and *Lernaea cyprinacea* were collected from five species. All five fish species had Monogenea (Gyrodactylidae and Dactylogyridae) in their skins and gills, the highest prevalence was observed in *C. auratus* and the lowest was in *P. scalare* and *S. discus*. Also, *Capillaria *sp. was reported as a first record from the abdominal cavity of *P. scalare* in Iran. Our findings revealed that the protozoal infections are very common among aquarium fishes. Although, no gross pathology was observed among infected fishes, but it is likely that in case of any changes in the environment, then parasitic infections could be harmful.

## Introduction

Aquarium fish trade is a very important sector all over the world.^1^ The global trade in ornamental fish, associated aquarium and pond accessories is more than 7 × 10^9^ USD each year. They are a significant source of overseas benefit for many rustic communities in Africa, south America and south-east Asia.^1^ Thousands types of aquarium fish (commonly, poeciliids, guppy and cichlids) are collected and maintained by hobbyists.^1^ The biggest portion of the aquarium fish industry is the freshwater aquarium fish sector. Cultivation and propagation of ornamental fishes have been increased in the recent decades in Iran, for its beautiful appearance, the small size and easy maintenance.^1^

Although this worldwide interest in ornamental fish has led to development in their cultivation techniques, there are still many difficult-to-culture species with high demand. Ornamental fish pathogens spread very rapidly in the world because of their commercial benefits. Consequently, routine infectious disease controls are very important for risk analysis and precaution steps. Parasites are harmful and limiting factors in breeding and rearing ornamental fish industry.^2^ From economic aspects, parasitic diseases in fish have a particular importance, because of causing sterility, discoloration, change of body shape and decreased growth and weight of fish.^3^ Therefore, knowledge about fish parasites is crucial for successful aquaculture. For this reason, we aimed to isolate and identify the parasitic fauna of five species of ornamental freshwater fish in northern Iran.

## Materials and Methods

A total number of 400 apparently healthy ornamental fishes including Goldfish (*Carassius auratus*; n = 80), guppy (*Poecilia reticulate*; n = 80), angelfish (*Pterophyllum scalare; *n = 80), discus (*Symphsodon discus; *n = 80) and sailfin mollies (*Poecilia latipinna*; n = 80) were obtained from local ornamental fish farms in Mazandaran province (North of Iran) between 2011-2012, (Table 1). Live fishes were transferred to fish diseases laboratory at the Caspian Sea Ecology Research Center using portable air pump. 

The external surface, abdominal cavities and digestive tracts were examined for presence of parasitic fauna. Fish were first examined for ectoparasites using wet mount under a light microscope (Olympus, Tokyo, Japan).^1^ Then, the alimentary ducts of fish were observed under light and stereo microscope. Parasites of alimentary tracts were counted and fixed in 70% ethanol, and for examination, they were cleared using glycerine.^2^ Identification of the parasites was carried out using the identification keys.^2^^,^^4^^,^^5^

**Table 1:** The geographical distribution of sampling in each examined fish species in the Mazandaran province, Iran

**Fish species**	**Region/Location of sampling**
***Pterophyllum scalare***	Sari, Tonekabon
***Carassius auratus***	Babolsar, Amol, Sari
***Symphsodon discus***	Feridonkenari, Tonekabon
***Poecilia latipinna***	Joibar, Sari, Babol
***Poecilia reticulata***	Tonekabon, Sari, Babolsar

## Results

**Fig. 1:**
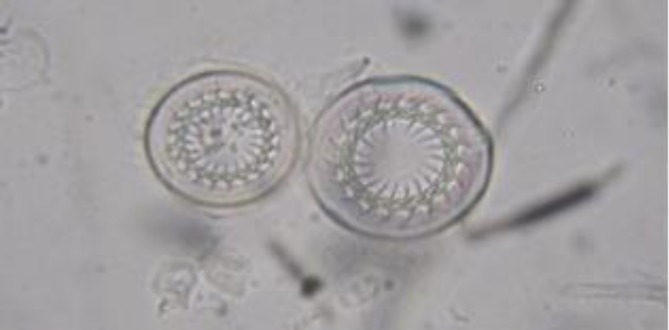
*Trichodina reticulata* isolated from discus (400×).

**Fig. 2:**
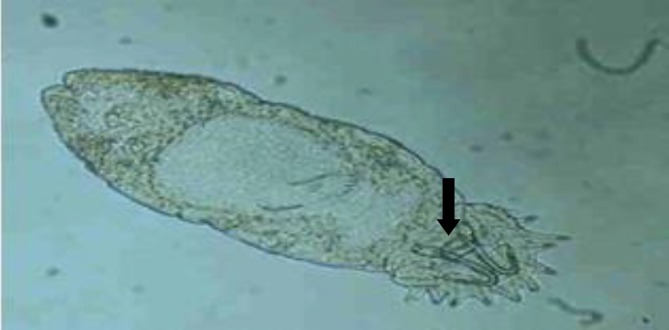
Two pairs of anchor hooks (arrow) of *Gyrodactylus *sp. isolated from guppy (100 ×).

**Fig. 3:**
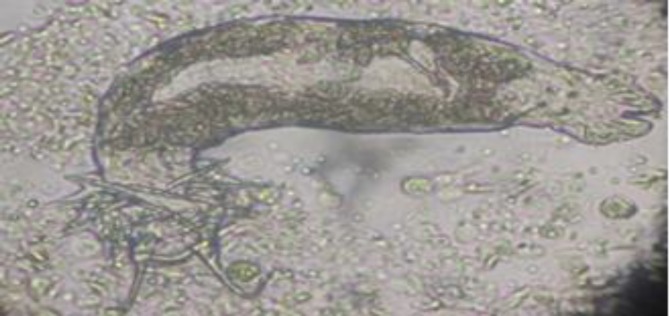
Anchors of *Dactylogyrus *sp. (Total length of anchor = 48.5 μm) isolated from Goldfish (100 ×).

**Fig. 4:**
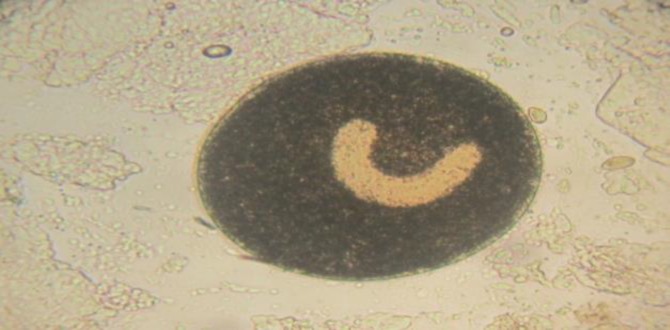
Large horseshoe-shaped macronucleus of *Ichthyophthirius multifiliis* isolated from discus (100 ×).

**Fig. 5:**
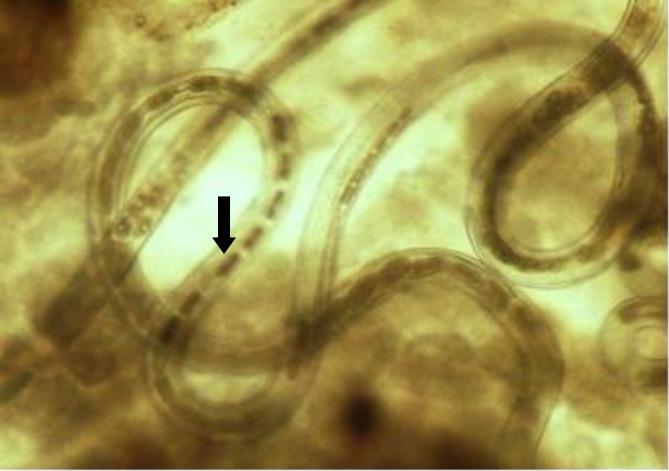
A female of *Capillaria *sp. with barrel-shaped eggs (arrow) in intestine of angelfish (100×).

During the sampling, the water temperature was 25 ± 3 ˚C, dissolved oxygen was 4.60 ± 0.50 mg L^-1^ and pH was 7.20 ± 0.60, respectively. Of all examined fishes, 380 fishes (95.00%) were infected by at least one parasite. One nematode (*Capillaria *sp.), two protozoa (*I. multifilis *and*, Trichodina reticulata*), two monogeneans (*Dactylogyrus *sp. and* Gyrodactylus *sp.) and one Crustacea (*L. cyprinacea*) were identified (Figs. 1 to 5 and Table 2). The hemorrhagic areas on the skin and gills, fins bleeding, scales losing and fin rot was observed in infected fish.

**Table 2:** Parasitic fauna in ornamental fish in Mazandaran province according to this study.

**Host**	**Parasites**	**Infected organ**	**Infected fish (%)**	**Range of infestation/Infection**
***Pterophyllum scalare***	*Dactylogyrus *sp. *Gyrodactylus *sp. *Ichthyophthirius multifiliis* *Trichodina reticulata * *Capillaria *sp.	Gills Skin Skin Skin/Fin Intestine	35.00 5.00 15.00 25.00 22.50	1-4 1-4 1-11 1-6 1-3
***Carassius auratus***	*Dactylogyrus *sp. *Gyrodactylus *sp. *Ichthyophthirius.multifiliis* *Trichodina reticulate* *Lernaea cyprinacea*	Gills Skin Skin Skin Skin/Fin	28.75 72.50 87.50 20.00 30.00	1-4 1-8 1-16 1-14 1-3
***Symphsodon discus***	*Dactylogyrus *sp. *Gyrodactylus *sp. *Ichthyophthirius multifiliis* *Trichodina reticulate*	Gills Skin Skin/Fin Skin/Fin	7.50 13.75 10.00 6.25	1-2 1-2 1-8 1-4
***Poecilia latipinna***	*Dactylogyrus *sp. *Gyrodactylus *sp. *Ichthyophthirius multifiliis* *Trichodina reticulata * *Capillaria *sp. *Lernaea cyprinacea*	Gills Skin Skin/Fin Skin/Fin Intestine Skin/Fin	16.25 28.75 12.50 12.50 1.25 5.00	1-2 1-5 1-4 1-7 1-2 1
***Poecilia reticulata***	*Dactylogyrus *sp. *Gyrodactylus *sp. *Ichthyophthirius multifiliis* *Trichodina reticulata * *Capillaria *sp. *Lernaea cyprinacea*	Gills Skin Skin/Fin Skin Intestine Skin/Fin	17.5 21.25 6.25 15.00 2.50 1.25	1-3 1-2 1-6 1-9 1 1-2

## Discussion

During the previous decades, fish parasites identification have become increasingly visible , because of the growth of freshwater ornamental fish industries throughout the world.^2^ Parasitic diseases affect physiologic and biologic characteristics, caused mechanical damage and economic losses in ornamental fish industries.^2^

Different parasite species were reported from various ornamental fish species around the world. *Tetrahymena *sp. was collected from gills of *Carnegiella strigata*, *Piscinoodinium pilullare *from the skin of *Carnegiella martae, Trichodinids* spp. from the skin of *C. strigata*, also *Nannostomus and* *Procamallanus *sp. was isolated from the intestine of *Paracheirodon axelrodi*.^8^ Koyun reported* Gyrodactylus katharine *and *Gyrodactylus carassii *from the gills of *C. carassius*,^9^ *Ichthyobodo *sp., *I. multifiliis*, *Chilodonella *sp., *Trichodina *spp from the skin., *Dactylogyrus extensus*, *Gyrodactylus bullatarudis*, *L. cyprinacea*, *Argulus foliaceus*, *Argulus japonicus *and *Capillaria *sp. from the external parts of goldfish, guppy and cichlids.^10^ *Ambiphyra *spp. was reported from the skin of guppy,^11^ and also, *Oodinium pillularis *was isolated from the skin of Poecilidae.^12^

In Iran, there were also many reports of parasite fauna from ornamental fishes for example, Meshgi *et al.* reported *Dactylogyrus rotator*, *Chiloldonella* sp., *Hexamita *sp*., Ictyobodo necator, I. multifiliis, Microsporidium,* *Myxosporida* sp., *Tricodina *spp., and *L. cyprinicea *from Aquarium fishes around Tehran.^1^

*Ichthyophthirius* *multifiliis, Gyrodactylus *sp., *Dactylogyrus* sp.*, Trichodina *spp., *Argulus coregoni*, *A. japonicas*,* A. foliaceus* was reported from *C. auratus.*^13^ Also, *I. multifiliis,* *Dactylogyrus* sp.*,* *Microsporidian *sp. and *Ichthyobodo *sp. were reported from angelfish in the Mazandaran province.^14^

In this study, *I. multifiliis* had the highest infection rate in *C. auratus*. The highest prevalence of Gyrodactylidae and Dactylogyridae were observed in *C. auratus* and the lowest in *P. scalare* and *S. discus*, respectively. 

In our study, *Capillaria *sp. was reported for the first time from *P. scalare *in Iran. This nematode may cause high mortality in aquarium fishes. Rahmati-holasoo *et al.* showed that infection with* Capillaria *sp. could cause a great loss in ornamental fish from Cichlidae in Iran. ^15^

It seems that many factors such as water quality, fish density, diet, physiology of host and parasite life cycle may have contributed to the severity and type of these parasites.^2^ Given the important role of risk factors, reducing stressful situations through improved management and environmental conditions such as improved water quality and switch on time, reduction of organic matter, avoiding excessive density of fish and unnecessary manipulation and using appropriate disinfectants in farms can be useful to control and reduce economic losses caused by parasitic disease in ornamental fishes. 

The identified parasites in this study have not been reported as a parasitic problem in Iran. However, the rate of infection in these aquarium fishes was low. The possibility of transmition of contamination to the native aquarium fishes, even farmed fishes should be taken into consideration.
